# Role of 1% alendronate gel as adjunct to mechanical therapy in the treatment of chronic periodontitis among smokers

**DOI:** 10.1590/1678-7757-2016-0201

**Published:** 2017

**Authors:** Anuj SHARMA, Achala RAMAN, Avani Raju PRADEEP

**Affiliations:** 1 Dental Pearl, Jharkhand, India.; 2 Government Dental College and Research Institute, Department of Periodontics, Bangalore, Karnataka, India.

**Keywords:** Alendronate, Chronic periodontitis, Smoking, Regeneration

## Abstract

**Objective:**

Alendronate (ALN) inhibits osteoclastic bone resorption and triggers osteostimulative properties both in vivo and in vitro, as shown by increase in matrix formation. This study aimed to explore the efficacy of 1% ALN gel as local drug delivery (LDD) in adjunct to scaling and root planing (SRP) for the treatment of chronic periodontitis among smokers.

**Material and Methods:**

75 intrabony defects were treated in 46 male smokers either with 1% ALN gel or placebo gel. ALN gel was prepared by adding ALN into carbopol-distilled water mixture. Clinical parameters [modified sulcus bleeding index, plaque index, probing depth (PD), and periodontal attachment level (PAL)] were recorded at baseline, at 2 months, and at 6 months, while radiographic parameters were recorded at baseline and at 6 months. Defect fill at baseline and at 6 months was calculated on standardized radiographs by using the image analysis software.

**Results:**

Mean PD reduction and mean PAL gain were found to be greater in the ALN group than in the placebo group, both at 2 and 6 months. Furthermore, a significantly greater mean percentage of bone fill was found in the ALN group (41.05±11.40%) compared to the placebo group (2.5±0.93%).

**Conclusions:**

The results of this study showed 1% ALN stimulated a significant increase in PD reduction, PAL gain, and an improved bone fill compared to placebo gel in chronic periodontitis among smokers. Thus, 1% ALN, along with SRP, is effective in the treatment of chronic periodontitis in smokers.

## Introduction

Bacterial biofilm has been considered one of the main aetiological factors in periodontal diseases^10^. Additionally, extensive researches suggest that host-derived enzymes, cytokines, and other mediators play a direct role in extracellular matrix (ECM) destruction in periodontitis^20^. The bacteria, therefore, initiate disease by activating host mechanisms that then destroy the supporting structures of the periodontium. This justifies how initiation and progression of periodontal diseases can be inhibited by interfering with host factors.

Conventionally, periodontal disease is treated by mechanical periodontal therapy, but certain cases may require adjunctive chemical periodontal therapy. Chemical periodontal therapy may involve various host modulators for controlling periodontal tissue destruction.

Bisphosphonates (BPs) are carbon-substituted pyrophosphate (P-C-P) analogs that include potent inhibitors of bone resorption, which have been effectively used to control osteolysis or reduce bone loss in Paget’s disease, metastatic bone disease, hypercalcemia of malignancy^1^, and osteoporosis^16^.

Alendronate sodium (ALN) is an amino bisphosphonate and, once taken up by bone, it acts as an antiosteolytic agent. ALN binds to resorption surfaces and is locally released during the acidification associated with osteoclastic activity. This release leads to a rise in the local concentration of ALN, resulting in an alteration in the ruffled border membrane characteristic of osteoclasts without destroying the cells. Therefore, ALN seems to have a potential to be used as an inhibitor of alveolar bone resorption in the treatment of periodontitis.

Previous studies have shown the effects of systemic ALN in human and other animal models in decreasing bone loss and increasing alveolar bone density^12,15,17^. Additionally, studies have observed that topical application of ALN was highly effective in reducing alveolar bone resorption after mucoperiosteal flap surgery^3,4,22,24,30^.

Evidence from cross-sectional and case-control studies in various populations and an abundant number of reviews on the subject have shown that adult smokers are about two to four times more likely to have periodontitis than nonsmokers. Previous studies indicated that smokers did not respond to non-surgical periodontal therapy^2,13^. Additionally, smokers showed less probing depth reduction and attachment gain, compared to nonsmokers, for periodontal surgical treatment^11,23^.

Considering the abovementioned facts, the current study is designed to evaluate the efficacy of 1% ALN gel as local drug delivery along with scaling and root planing (SRP) for the treatment of intrabony defects among smokers with chronic periodontitis.

## Material and methods

### Source of data

In this 6 month follow-up longitudinal interventional study, a total of 52 male smokers (age range: 30-50 years old) with chronic periodontitis was selected from the outpatient section of the Department of Periodontics, Government Dental College & Research Institute, Bangalore. The research protocol was presented to the Ethical Committee and Review Board of the institution. After ethical approval, all subjects were verbally informed and written informed consent was taken for participation in the research. The study was conducted from March 2010 to April 2011.

### Selection criteria

Systemically healthy subjects with probing depth (PD) ≥5 mm or periodontal attachment level (PAL) ≥4 to 6 mm and a radiographic vertical bone loss ≥3 mm with no history of periodontal therapy or use of antibiotics in the preceding 6 months were included in the study. Smoking history was collected by self-report after a standardized questionnaire. Subjects were classified as smokers and nonsmokers based on criteria established by the Centers for Disease Control and Prevention (CDC): “current smokers” were defined as those who had smoked 100 or more cigarettes over their lifetime and smoked at the time of interview; “former smokers” had smoked 100 or more cigarettes over their lifetime but were not currently smoking; and “nonsmokers” had not smoked 100 or more cigarettes over their lifetime. Former smokers were excluded in an attempt to make a clear discrimination between smokers and nonsmokers. Subjects with the following characteristics were excluded from the study: known systemic disease; known or suspected allergy to the ALN/bisphosphonate group; systemic ALN/bisphosphonate therapy; aggressive periodontitis; use of smokeless tobacco in any form; alcoholism; immunodeficiency.

**Figure 1:**
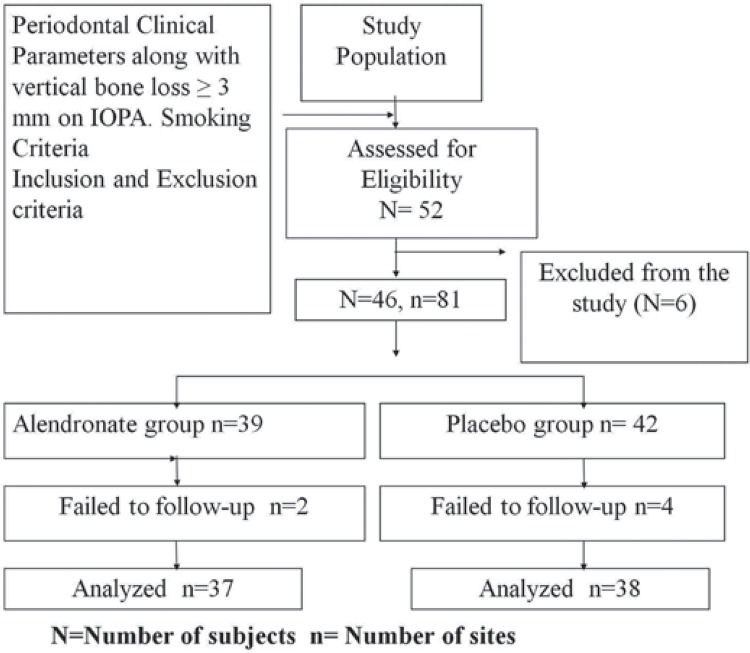
Study flow chart

46 subjects (out of 52 enrolled), who matched clinical and radiographic parameters, were recruited for double-blind clinical study (Figure 1). Multiple sites from the same patients were also considered in case of fulfilling selection criteria. A total of 75 sites were randomly (by computer generated system) assigned to either ALN or placebo group. 37 sites (2 failed) and 38 sites (4 failed) completed the study in the ALN group and placebo group, respectively. Another clinician (AS) treated subjects enrolled to either group. All pre- and posttreatment clinical parameters were recorded by an examiner (ARP) who was masked to the type of treatment received by the subjects, while another clinician (AS) provided treatment to both groups.

In the ALN group, sites were treated with SRP followed by 1% ALN gel (10 mg/ml) local drug delivery, while in the placebo group sites were treated with SRP followed by placebo gel placement. Subjects were blinded for allocation into ALN or placebo group. SRP was performed at baseline until the root surface was considered smooth and clean by the operator (AS). No antibiotics or anti-inflammatory agent were prescribed after treatment. Clinical parameters, including modified sulcus bleeding index (mSBI)^18^, full mouth and site-specific plaque score (PI)^28^, PD, and PAL, were recorded at baseline (before the SRP) and at 2 and 6 months. A custom-made acrylic stent and a no. 15 color-coded University of North Carolina periodontal probe were used to standardize the measurement of clinical parameters.

### Intra-examiner calibration

30 sites were examined twice for intra-examiner calibration. Calibrations were considered for measurements similar to 1 mm at the 95% level.

### Radiographic assessment of Intrabony Defects (IBD)

Bone fill was evaluated at baseline and at 6 months using an image analyzer (Scion image Corporation, Frederick, MA, USA). IBD was measured on the radiograph by measuring the vertical distance from the crest of the alveolar bone to the base of the defect. Individually customized bite blocks and a parallel-angle technique were used to obtain films as reproducible as possible. All radiographs were reviewed in a single reference center by a masked evaluator. For assessment, radiographs were scanned with a 6400 DPI scanner (Epson Perfection V700, Bangalore, India) by an evaluator who was blinded to the surgical procedure performed in the subjects. The radiographic IBD depth was measured by computer aided software program, as previously used^25^.

### Primary and secondary outcome measures

The primary outcome of the study was PD and PAL. The secondary outcomes included complete bone defect fill.

### Formulation of 1% ALN gel

ALN gel was prepared as described by Reddy, et al.^24^ (2005). Briefly, ALN (Apex Pharma, Ankleshwar, Gujarat, India) was dissolved in a required amount of distilled water to achieve 1% ALN concentration. A weighed quantity of carbopol 934P (2% w/w) was taken and added to the distilled water. The mixture was gradually stirred and carbopol was allowed to soak for 2 h. 1% triethanolamine was added to neutralize the carbopol solution and to form the gel. The pH was adjusted to 6.8. Finally, the required amount of methylparaben (0.1%) and propylparaben (0.05%) were dissolved in ethanol and added to the gel. The placebo gel was prepared by the abovementioned procedure without adding the active ingredient (ALN).

### Local drug delivery

The prepared ALN gel (10 mg/ml) was dispensed into the periodontal pockets with intrabony defects using a syringe with a blunt cannula. Patients were instructed not to use forceful brushing or interdental aids at the treated sites until the appointment after 2 months and to avoid chewing sticky or hard food.

### Statistical analysis

Power analysis calculations were performed before the study was initiated. To achieve 90% power and detect mean differences of the clinical parameters between groups, 30 sites in each group were required. The categorical variable (site-specific PI) was expressed as percentage and the continuous variable (Full mouth PI, mSBI, PD, PAL, and IBD depth), as mean ± standard deviation. Site-specific PI was compared by using Chi-squared test or Fisher’s exact test when the expected frequency was less than 5. Normality assumption was tested using Shapiro-Wilk’s W test. If the continuous variable followed a normal distribution, a comparison would be carried out in the treatment group using student’s t test. Statistical significance was defined as p<0.05. Statistical analysis was performed with SPSS version 15, SPSS Inc., Chicago, IL, USA.

## Results

46 subjects (multiple sites/subject) out of 52 completed the study (Figure 1). All subjects tolerated the drug well without any complications or adverse reactions. Soft tissues healed within normal limits, and no significant visual differences were noted.

**Figure 2:**
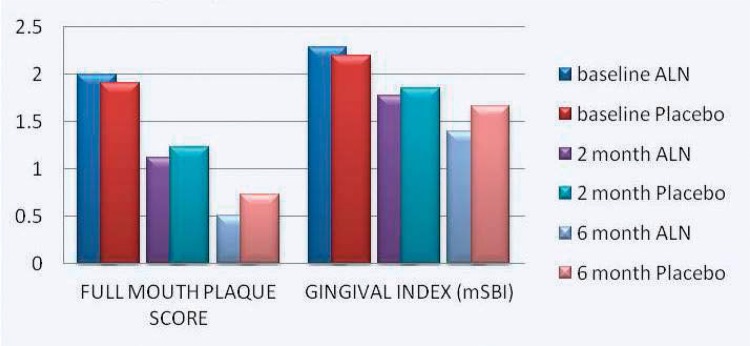
Full mouth plaque scores and gingival index for alendronate and placebo groups at different time intervals

Both groups showed improvement in site specific and full mouth PI score, but improvement was not statistically significant among both groups at any time point for site-specific PI. Improvement in full mouth PI score was significantly higher in the ALN group (p<0.05) compared to the placebo group at 6 months (Figure 2).

In both groups, mSBI showed no difference at baseline, at 2 months, and at 6 months (Figure 2).

Clinical parameters PD and PAL showed no difference in intergroup comparison at baseline and showed significant PD reduction and PAL gain at 2 and 6 months at p<0.05 (Tables 1, 2).

**Table 1:** Probing depth, periodontal attachment level, and intrabony defect depth in alendronate and placebo groups at different time interval

Parameters (mm)	Visits	ALN	Placebo	t-value†	p-value
Probing depth	Baseline 2 Month 6 Month	7.84 ±2.04 5.05 ±1.78 3.68 ±1.93	7.62 ±1.97 6.38 ±1.84 5.57±1.88	0.21 9.87 18.253	0.645 <0.002* <0.001*
Periodontal attachment level	Baseline 2 Month 6 Month	6.43 ±1.77 4.05 ±1.94 2.49 ±1.53	6.19 ±1.54 5.22 ±1.73 4.41±1.83	0.4 7.36 23.79	0.531 <0.008* <0.001*
Intrabony defect depth	Baseline 6 Month	5.18 ±1.00 3.07 ±0.94	5.10 ±0.95 4.97 ±0.95	0.123 74.03	0.727 <0.001*

*Statistically significant at p<0.05

† t test

**Table 2:** Change in mean probing depth, periodontal attachment level, and intrabony defect and percentage change in bone fill in alendronate and placebo groups from baseline

Clinical parameters	% change from baseline	ALN group	Placebo group	t- value†	p- value
Mean PD	2 Month 6 Month	2.78 ± 1.20 4.16 ± 1.23	1.24± 0.83 2.05± 0.94	41.01 68.1	<0.001* <0.001*
Mean PAL (mm)	2 Month 6 Month	2.38 ± 0.95 3.95 ± 0.88	0.97 ± 0.92 1.78 ±1.22	41.317 75.789	<0.001* <0.001*
Mean IBD (mm)	6 Month	2.10 ± 0.69	0.12 ± 0.04	302.01	<0.001*
Bone defect fill	6 Month	41.05 ±11.40	2.5 ± 0.93	420.07	<0.001*

*Statistically significant at p<0.05

† t test

Radiographic parameter IBD showed statistically significant mean reduction of 2.10±0.69 mm in the alendronate group, in comparison to the placebo group (0.12±0.04 mm) (Table 2). Alendronate sites presented a significantly higher vertical defect fill (41.05±11.40%) than placebo sites (2.5±0.93%) at 6 months (Table 2).

## Discussion

This study has evaluated the clinical efficacy of 1% ALN gel along with SRP for the treatment of IBD in smokers with chronic periodontitis and showed significant radiographic bone fill and improvement in clinical parameters compared to placebo gel.

ALN and other ingredients of gel formulation are approved by the Food and Drug Administration (USFDA) for oral uses. Studies^24,26^ have explored different properties for various concentrations of ALN and found 1% ALN gel is the optimum dosage for LDD, thus 1% ALN was the choice for the current research.

ALN is a bisphosphonate that acts as a potent inhibitor of bone resorption. It is now generally accepted that the main cell by which bisphosphonates mediate their action is the osteoclast. Various mechanisms to be involved are inhibition of osteoclast recruitment, osteoclast adhesion, osteoclast activity, and shortening of osteoclast lifespan (apoptosis)^5^. Several reports have shown that bisphosphonates not only induce osteoblasts to secrete inhibitors of osteoclast-mediated resorption, but also stimulate the formation of osteoblast precursors and mineralized nodules, thereby promoting early osteoblastogenesis^7^.

Histometric analyses showed more percentage of bone in the furcation area that was treated with 1 mL sodium ALN irrigation (10^-5^M), when compared to control groups of experimental periodontitis in rats at 7 and 15 days^3^. Immunohistochemical analyses also expressed stronger osteoprotegerin immunolabeling, weaker receptor activator of nuclear factor-kB ligand immunolabeling, and fewer tartrate-resistant acid phosphatase-positive cells in rats^3^. Collectively, this study showed signs of shift from inflammation to periodontal health.

To our knowledge, there have been no studies reporting the use of 1% ALN gel as LDD in the treatment of chronic periodontitis in smokers. Therefore, a direct comparison with other studies is not possible. Comparing changes in clinical and radiographic parameters with 1% ALN gel as the local drug delivery used in the treatment of subjects with CP and aggressive periodontitis (AgP) in previous studies^26,27^, the improvement in clinical parameters in this study was higher in AgP subjects but lower in subjects with CP, while radiographic bone fill was higher in CP subjects but lower in AgP subjects. PD reduction was lower (4.16±1.23) in CP subjects (4.48±1.27 mm), as reported in previous study^26^, but greater compared to AgP subjects (3.88±1.39 mm) at 6 months, as observed in previous study^27^. Similarly, PAL gain was lower (3.95±0.88) in CP subjects (4.03±0.84 mm), but greater compared to AgP subjects (3.27±1.11 mm) at 6 months. Conversely, percentage change in bone fill was nearly equal but lower (41.05±11.40) in AgP subjects (46.1±9.48%) and nearly equal but greater in CP subjects (40.4±11.7%)^26,27^.

The results of our study are in line with previous studies that have reported that ALN was highly effective in reducing alveolar bone resorption following mucoperiosteal flap surgery^24,30^. This study found a percentage of IBD fill in the ALN group of 41.05±11.40%, compared to 2.5±0.93% in the placebo group at 6 months. This is in accordance with our previous studies^26,27^. ALN LDD also showed significant percentage of bone fill (40.87±2.73%) compared to atorvastatin LDD (34.61±5.11%) in a recent clinical trial^22^. Considering the abovementioned facts and results of previous studies, it can be proposed that direct subgingivally placed ALN will be a better approach for periodontal healing of bony defects in smokers with chronic periodontitis. The probable mechanism for better bone fill in the current study can be explained by the fact that tobacco components have shown to have direct effects on certain bone resorptive mediators. Tappia, et al.^29^ (1995) reported that smokers exposed to bacterial lipopolysaccharide had significantly higher plasma levels of TNF alpha and IL-6 than nonsmokers, while other study reported that abrogation of interleukin-6 production by alendronate in human osteoblastic cells can occur, which could also affect osteoclastic activity^8^.

This study has considered the technique of subgingivally delivering ALN directly into pockets of smokers with chronic periodontitis as the local drug delivery systems that offers the advantages of high concentrations at the target site with reduced dosage, fewer applications, and high patient acceptability^9^. Compared to a systemic regimen, local delivery may offer important beneﬁts in terms of adverse reactions and patient compliance, as also reported in previous study^19,26,27^.

The current study reported presence of bone fill after 6 months from baseline, while ALN was found to be present in gingival crevicular fluid sample only at 1 month and absent at 2 months, as reported in a previous study^26^. This can be explained by the release of BPs from bounded bone mineral during bone resorption by osteoclasts. This could lead to a localized accumulation of BPs, which could directly perturb osteoclastic activity or indirectly target osteoblasts and macrophages, resulting in decreased osteoclastic chemotaxis and activity^6^. Carbopol was used as vehicle for preparation of ALN gel in this study, which is considered to provide intimate contact and prolong the residing time of a dosage form in the periodontal pocket, by specific interfacial forces in a process commonly referred as mucoadhesion, after LDD^14,21^.

## Conclusion

This study showed 1% ALN gel caused significant improvement in periodontal clinical parameters in smokers with chronic periodontitis compared to control subjects. 1% ALN can be used as a local drug delivery system along with SRP for nonsurgical management of periodontitis.
